# Persistent delirium in older hospital patients: an updated systematic review and meta-analysis

**DOI:** 10.56392/001c.36822

**Published:** 2022-08-09

**Authors:** Jonathan Whitby, Anita Nitchingham, Gideon Caplan, Daniel Davis, Alex Tsui

**Affiliations:** 1 MRC Unit for Lifelong Health and Ageing, University College London; 2 The Prince of Wales Clinical School, University of New South Wales

**Keywords:** persistent delirium, systematic review, meta-regression, older people

## Abstract

**Introduction:**

Delirium is associated with future dementia progression. Yet whether this occurs subclinically over months and years, or persistent delirium merges into worsened dementia is not understood. Our objective was to estimate the prevalence of persistent delirium and understand variation in its duration.

**Methods:**

We adopted an identical search strategy to a previous systematic review, only including studies using a recognised diagnostic framework for ascertaining delirium at follow-up (persistent delirium). Studies included hospitalised older patients outside critical and palliative care settings. We searched MEDLINE, EMBASE, PsycINFO and the Cochrane Database of Systematic Reviews on 11th January 2022. We applied risk of bias assessments based on Standards of Reporting of Neurological Disorders criteria and assessed strength of recommendations using the grading of recommendation, assessment, development and evaluation (GRADE) approach. Estimates were pooled across studies using random-effects meta-analysis, and we estimated associations with follow-up duration using robust error meta-regression.

**Results:**

We identified 13 new cohorts, which we added to 10 from the previous systematic review (23 relevant studies, with 39 reports of persistent delirium at 7 time-points in 3186 individuals admitted to hospital care (mean age 82 years and 41% dementia prevalence). Studies were mainly at moderate risk of bias. Pooled delirium prevalence estimates at discharge were 36% (95% CI 22% to 51%, 13 studies). Robust error meta-regression did not show variation in prevalence of persistent delirium over time (-1.6% per month, 95% CI -4.8 to 1.6, p=0.08). Margins estimates for this model indicate a prevalence of persistent delirium of 16% (95% CI 6% to 25%) at 12 months.

**Conclusions:**

This systematic review emphasises the importance of delirium as a persistent and extensive problem (GRADE certainty = moderate), raising questions on chronic delirium as a clinical entity and how it might evolve into dementia. Addressing persistent delirium will require a whole-system, integrated approach to detect, follow-up and implement opportunities for recovery across all healthcare settings.

## Introduction

Delirium is particularly prevalent among older people and is associated with significant adverse outcomes, including long-term cognitive decline and increased mortality.^1^ Several clinical guidelines and standards address its detection, prevention and management in hospitals.^2–6^ Each of these recognises that questions remain over the natural history of delirium, particularly in its relationship with dementia.^7^ Although greater brain atrophy is associated with prolonged delirium, and incident delirium with future dementia progression,^8–10^ whether underlying dementia leads to a more severe or prolonged delirium remains unknown.^11,12^ In addition, the course and timeframe over which delirium-associated cognitive decline occurs, either stepwise or insidiously over months and years, or how persistent delirium symptoms phenotypically transform into worsened dementia, is not understood.

Persistent delirium is both a challenge to health care systems and a research opportunity.^13^ First, there is no current clinical or academic consensus on how long delirium has to be present to be described as *persistent*. Defining start and end points of delirium is already challenging, particularly in the context of pre-existing dementia, when overlapping clinical features reduce specificity of both delirium and dementia screening instruments.^8^ This potentially delays delirium recognition and prolongs it. Moreover, there is no agreement on the time between temporal clusters of delirium symptoms that would constitute a single episode. Over-all, our understanding of *how* delirium resolves remains limited: do clinical features improve as a single entity or do deficits resolve at varying rates? Are resolution patterns heterogeneous among different patients with divergent cognitive prognoses?

Persistent delirium has been of academic interest since a systematic review from 2009 suggested one in five cases were still evident six months after discharge.^14^ Subsequently, the number of older people presenting for urgent and emergency care has increased.^15^ There has also been a consistent trend for more acute presentations of people living with dementia; around half are admitted within the first 12 months of diagnosis.^16^ In light of these changes, we set out to update the systematic review to provide current estimates for the prevalence of persistent delirium and understand variation in its duration. For this review, persistent delirium was defined as an accepted diagnosis of delirium during admission and at follow-up, performed at least one week after initial assessment.

## Methods

### Eligibility Criteria

We followed the 2020 PRISMA guidance.^17^ We used the same criteria applied in the previous systematic review: (i) study population of at least 20 hospital patients; (ii) patients aged ⩾50 years; (iii) prospective study with follow-up of at least one week; (iv) acceptable definition of delirium at enrolment.^14^ Only studies published in English or French were included. Studies investigating delirium in critical care and in the context of terminal illness or palliative care were excluded: we deemed delirium in these groups was likely to have been driven by setting specific precipitants, such as intubation, central venous access, anaesthesia and sedative medications, resulting in a population distinct from ward-based patients.

Our only modification required studies to use a recognised diagnostic framework for ascertaining delirium at follow-up (*persistent delirium*); studies from the original systematic review meeting this criterion were carried over into the current analysis. Given this was an update of a previous systematic review, we did not devise a *de novo* protocol for PROSPERO. Risk of bias assessments were based on Standards of Reporting of Neurological Disorders criteria and strength of recommendations were assessed using the grading of recommendation, assessment, development and evaluation (GRADE) approach.^18,19^

### Outcome Measures

The *a priori* defined primary outcome was proportion of patients with delirium at follow-up, where the denominator was the number of participants who had delirium at inception, extracted as prevalence percentages. We considered any definition based on the Diagnostic and Statistical Manual (DSM), International Classification of Disease, the Confusion Assessment Method (CAM) or the Delirium Index to be sufficiently detailed to ascertain delirium reliably.^20,21^

### Search Strategy

Updating the original review, we searched from 1 year before the previous end date (September 2006) to 11^th^ January 2022. We searched the same electronic databases: MEDLINE, EMBASE, PsycINFO and the Cochrane Database of Systematic Reviews, using the following search terms: Delirium [Title] AND (prognosis OR outcome OR aged OR occurrence) [Title/Abstract], replicating the original search strategy. We confirmed the sensitivity of the search by ensuring that we captured all studies identified by the previous review when we applied these terms to that review’s timeframe.

### Data Collection And Study Selection

Covidence (www.covidence.org, Veritas Health Innovation Ltd.) was used to manage the abstract and full-text screening, assessing risk of bias and data extraction (including study characteristics, ascertainment methods, delirium prevalence at each time point, study-level mean age, study-level dementia prevalence). We also documented the duration of the delirium – usually the length of stay – where this was reported for persistent delirium at discharge. Three researchers independently reviewed titles and abstracts (J.W., A.N., A.T.) to determine the eligibility. Conflicts were resolved by discussion and consensus. The same reviewers extracted data using a *pro forma*.

### Assessment Of Quality And Biases

Using the Standards of Reporting of Neurological Disorders criteria to determine risk of bias, we considered bias arising (i) from specific patient settings, e.g., general medical patients, cohorts with intracerebral haemorrhage; (ii) from sample selection, e.g., convenience, consecutive; (iii) from sample criteria, e.g., excluding patients unable to consent, assessed as being too sick; (iv) from reference standard used, e.g., DSM, CAM; (v) from expertise in applying the reference standard, e.g. routine data, dedicated researcher.^19^ We rated studies *high*, *low* or *unclear* risk of bias in each of the five domains, graphically representing these using the *robvis* app.^22^ Strength of evidence was assessed using the GRADE framework, which takes into account risk of bias, inconsistency, indirectness, imprecision and publication bias.^18^

### Statistical Analysis

We extracted prevalence of persistent delirium at each time point from within each study and calculated their standard errors (sqrt [p (1-p) / n)] and 95% confidence intervals. We assumed methodological heterogeneity across studies, accounting for this heterogeneity using DerSimonian–Laird random-effects models.^23^ Statistical heterogeneity was assessed with the I^2^ statistic. To assess publication bias, we plotted the estimated proportion of delirium occurrence against the standard error of that estimate and inspected the degree of asymmetry.

Our preliminary forest plots stratified the pooled data by length of follow-up. However, we found the estimates for *persistent delirium at discharge* reported a wide range of inpatient delirium durations (up to 30 days for one study^24^). Therefore, we corrected the reported follow-up duration to account for this initial difference. Where studies did not report initial delirium duration or length of stay (n=4), or they were conducted in a post-acute setting (n=3), we imputed the median delirium duration (6 days).

Meta-regression was used to estimate the relationship between persistent delirium and follow-up time, mean age (the commonest reported metric, mean age otherwise calculated from published summary statistics), and dementia prevalence in each study sample. Standard errors were estimated using permutations based on a Monte Carlo simulation procedure to account for clustered observations, where studies reported serial prevalence in the same cohort.^25^ To further address the non-independence of these longitudinal observations, we used robust errors meta-regression to give summary estimates for persistent delirium over time.^26^ We used Stata 17.0 (StataCorp, Texas) for all analyses.

## Results

**Figure 1:**
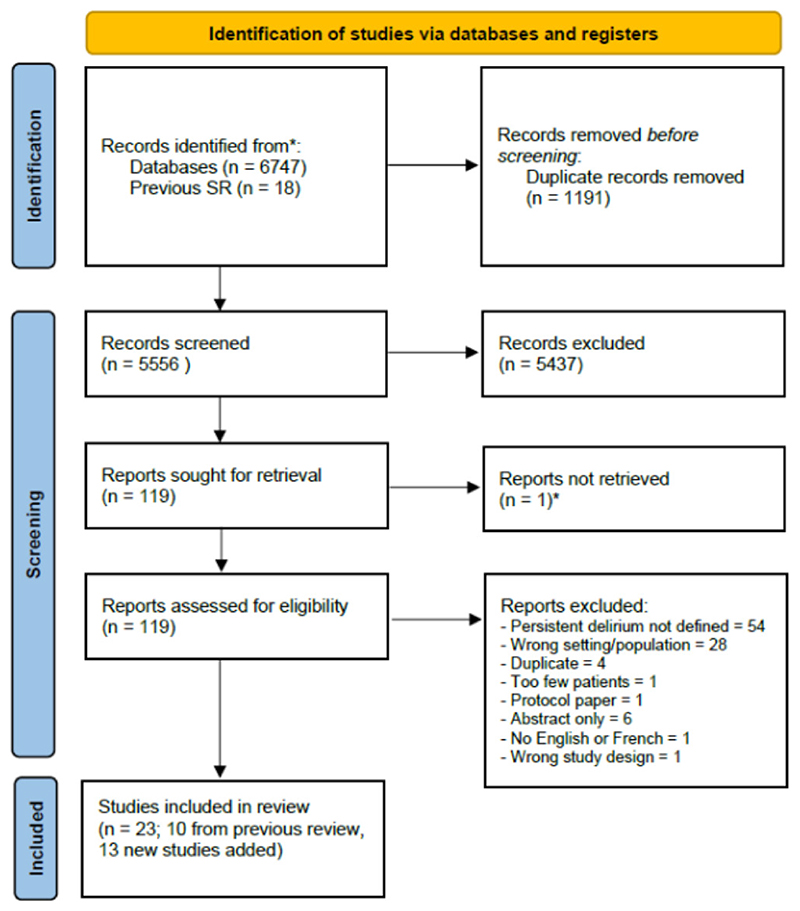
PRISMA flow diagram detailing study selection process. * One study included in original systematic review could not be directly retrieved, but it was possible to use data secondary reports. Reasons for exclusion at full

We identified 6474 articles, screening 5556 after removing duplicates (Figure 1). We assessed 119 full-text articles for eligibility. We identified 13 new cohorts, which we added to 10 from the previous systematic review, giving a total of 23 relevant studies with 39 reports of persistent delirium at 7 time points in 3186 individuals (Table 1).^24,27–48^ Most studies were in medical or surgical patients (14 medical; 9 surgical; 3 post-acute care; 2 stroke); all were from high-income settings. Case mix by ethnicity was not generally reported. Samples ranged in size (n=23 to n=590) and duration of follow-up (up to 18 months, 1 study). Mean age and proportion with dementia were 82 years and 41%, respectively, though both varied substantially between different study cohorts. Most studies either did not report details of dementia ascertainment or based it on a diagnosed medical history. However, two studies used the Blessed dementia scale^33^ or a follow-up assessment at 3 months to determine whether DSM-IV-TR criteria for pre-index dementia could be retrospectively diagnosed.^44^ One article from the original systematic review could not be directly accessed despite extensive archival searches,^28^ so we used secondary data reported in that review with appropriate caution in our risk of bias assessments for that study.

In the main, studies were at low risk of bias insofar as most followed a sample representative of the target setting (e.g., acute hospital care) and used consistent outcome ascertainment instruments. Around half excluded patients unable to give consent (and did not report procedures to allow proxy consent) and/or excluded participants too sick to assess for delirium (GRADE certainty rating for risk of bias = *moderate*) (Supplementary Figure 1). Case ascertainment was based on a consensus definition (e.g. DSM) in 9 studies; the rest used instruments such as CAM. Given each DSM definition has different degrees of restrictiveness,^49^ the GRADE certainty rating for indirectness was *moderate*.

**Figure 2:**
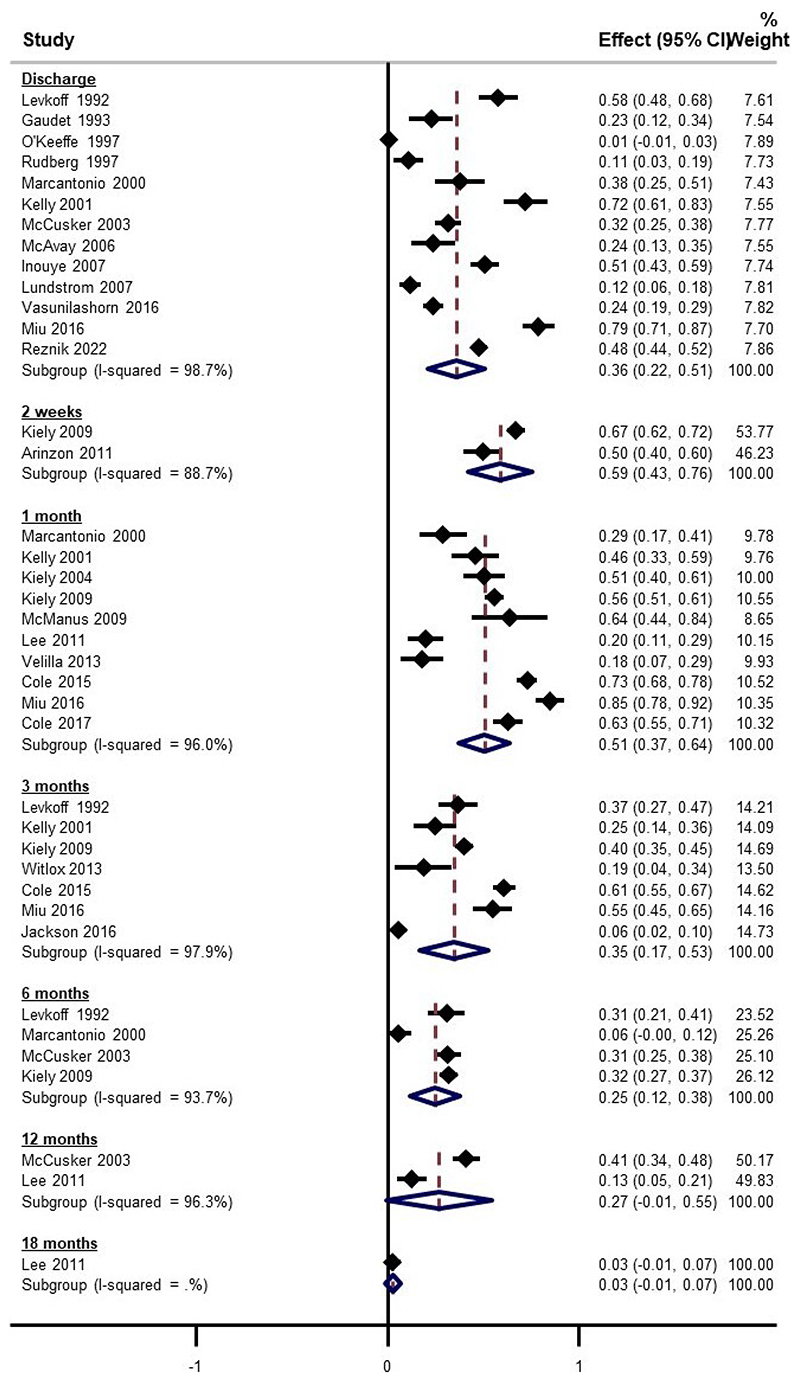
Forest plot showing pooled estimates of persistent delirium, stratified by study follow-up period. NOTE: Weights are from random-effects model

Pooled estimates for delirium prevalence at discharge was 36% (95% CI 22% to 51%, 13 studies) (Figure 2). Although there were few studies with data beyond 6 months, each time point reported considerable persistent cases (pooled estimates ranging from 3% to 59%). There was substantial statistical heterogeneity at all time points (all subgroup I^2^ >89%). Funnel plots of prevalence versus the standard error of the estimate did not demonstrate any asymmetry, leading to a GRADE certainty rating for lack of publication bias as *high* (Supplementary Figure 2).

**Figure 3:**
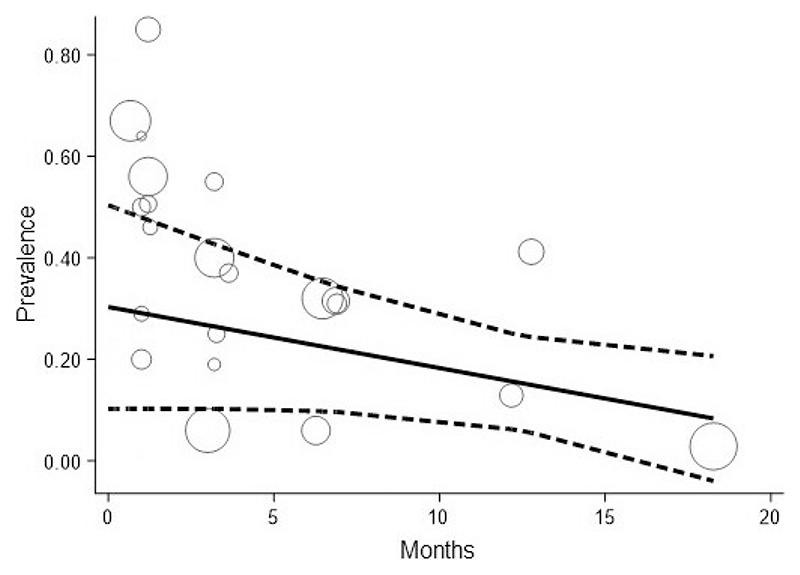
Robust error meta-regression showing estimated prevalence of persistent delirium over time. Solid line shows fitted meta-regression, with 95% CI (dashed lines). Circles are study estimates, proportional to sample size (inverse variance weighting).

Robust error meta-regression did not show variation in prevalence of persistent delirium over time (-1.6% per month, 95% CI -4.8 to 1.6, p=0.08) (Table 2, Figure 3). The lack of monotonic decrease in prevalence over time led to a GRADE certainty rating for inconsistency as *moderate*. Older study sample age was not associated with higher prevalence of persistent delirium (1.5% per year, 95% CI -0.6% to 3.6%, Table 2). Persistent delirium did not vary with the proportion of study sample with dementia, though only 16 studies reported this (Table 2). Figure 3 shows the predicted robust error meta-regression for change in prevalence over time. Margins estimates for this model indicate a prevalence of persistent delirium of 16% (95% CI 6% to 25%) at 12 months (Figure 3). The GRADE certainty for rating for precision was *low*.

## Discussion

We showed that though individual estimates of prevalence differ substantially, persistent delirium can remain a problem well beyond the acute phase and indeed may never recover. This persistence does not appear to vary with a prior diagnosis of dementia, but the study-level ecological measure of dementia was unlikely to account for undiagnosed cognitive impairment. Neither was a clear association evident with mean age of the study sample. Taken together, these findings provide evidence for a chronic form of delirium that may merge with underlying dementia. Either way, there appears to be a considerable need to focus delirium recovery for both individuals and health services.

Our data should be considered in light of a number of limitations. Other than duration, the variables we could use for meta-regression relied on study-level data on mean sample age and dementia prevalence. Mean age might not have been the most appropriate summary measure in older cohorts. Interpreting meta-regression estimates may be limited due to residual confounding for quantities such as frailty. Most studies had significant attrition due to mortality and loss to follow-up, though this might be expected to underestimate the true prevalence of persistent delirium. All included studies were performed in high-income countries, while all but one cohort involved acutely unwell patients, making it difficult to generalise our findings to other lower-income, community or elective settings. Our findings also do not apply to critical care or palliative care, specifically being unable to describe delirium in the terminal phase. Many studies only included delirium detected in the first 24-48 hours of admission, which could have led to under-ascertainment. We addressed the degree of overlap between the reports of *delirium at discharge* and the earlier follow-up time points (*2-weeks* and *4-weeks*) by using exact intervals where possible. Some publications only reported persistent delirium at discharge, and it is impossible to know how long these patients remained as cases. Similarly, it is unclear how long delirium might have been present before transfer for individuals admitted to post-acute care. For longer follow-up intervals, it would not have been possible to ascertain whether any incident delirium was persistent from the index event, or whether researchers were observing a second distinct episode of delirium. There was heterogeneity in delirium ascertainment measures. We also restricted our search to studies published in English or French, though only excluded one article based on publication language.

In updating the original systematic review with 15 years’ worth of new studies, we have extended those findings to demonstrate persistent delirium remains a significant possibility for around one in eight older patients after 12 months. We could not identify risk factors for persistent delirium, nor was there consistent information to describe if clinical findings evolved into a dementia syndrome. More broadly, these data are consistent with terminal decline in cognition observed in longitudinal studies.^50^ Higher education is associated with delayed onset of dementia, though a faster trajectory once terminal decline begins.^51^ Though previous cohorts have not considered delirium to be a driver, the Delirium and Population Health Informatics Cohort has shown that the largest post-delirium decline in cognition occurs in those with *higher* baseline cognition.^52^ Dedicated prospective studies are needed to fully capture the influence of evolving dementia reciprocally affecting persistent delirium.

Persistent delirium offers a unique opportunity to better understand the pathophysiological mechanisms underlying delirium. We postulate the optimum window might occur when the patient remains delirious, but the initial medical or surgical insult has resolved, perhaps pragmatically defined as normalisation of laboratory abnormalities, healed surgical wounds and beyond the active lifespan of newly administered potentially delirium-culprit medications. Laboratory and neuroimaging investigations can be compared with features already present in patients with dementia, identifying any differing or additional pathophysiological mechanisms unique to delirium, not operating in patients with dementia alone.

The clinical implications of our findings indicate an urgent need to develop and evaluate delirium recovery and cognitive rehabilitation services. These services are almost non-existent, certainly out of proportion with the potential demand, though multicomponent interventions show promise.^39^ Recognising the opportunities for the emerging field of interface acute geriatrics would be an important starting point,^53^ and continued community-based management of delirium is likely to have an impact.^54^ This systematic review emphasises the key importance of delirium as a persistent and extensive problem. Addressing persistent delirium will require a whole-system, integrated approach in order to detect, follow-up and implement opportunities for recovery across all healthcare settings.

## Supplementary Material

Prisma checklist

Supplementary information

## Figures and Tables

**Table 1:** Characteristics of included studies.

Study	Setting	N	Mean sample age (years)	Dementia (%)	Delirium ascertainment	Inpatient delirium duration (median)	Prevalence of persistent delirium						
							Discharge	2w	1m	3m	6m	12m	18m
Levkoff 1992^27^	M, S	91	81		DSM-III	19 days	*58%*			*37%*	*31%*		
Gaudet 1993^28^	M	52	85	39	DSM-IIIR	7 days	*23%*						
O’Keeffe 1997^24^	M	94	82	46	DSM-III	30 days	*1%*						
Rudberg 1997^29^	M, S	64	75	33	DSM-III-R	7 days	*11%*						
Marcantonio 2000^30^	S	52	79	66	CAM	5 days	*38%*		*29%*		*6%*		
Kelly 2001^31^	M	61	89		CAM	8 days	*72%*		*46%*	*25%*			
McCusker 2003^32^	M	181	83	70	DSM-III-R	18 days	*32%*				*32%*	*41%*	
Kiely 2004^33^	PAC	85	85	23	CAM	N/A			*51%*				
McAvay 2006^34^	M	55	80	27	CAM	15 days	*24%*						
Inouye 2007^35^	M	169	80	20	CAM	7 days	*51%*						
Lundstrom 2007^36^	S	129	82	32	DSM-IV	10 days	*12%*						
Kiely 2009^37^	PAC	412	84	38	CAM	N/A		*67%*	*56%*	*40%*	*32%*		
McManus 2009^38^	Stroke	23	75		CAM				*64%*				
Arinzon 2011^39^	M	92	80	90	CAM, DRS	16 days		*50%*					
Lee 2011^40^	S	70	80	10	CAM				*20%*			*13%*	*3%*
Velilla 2012^41^	M	45	87		DSM-IV				*18%*				
Witlox 2013^42^	S	27	84		CAM					*19%*			
Cole 2015^43^	M, S	278	85	55	CAM				*73%*	*61%*			
Jackson 2016^44^	M	125	84	36	DSM-IV					*6%*			
Miu 2016^45^	PAC	89	84		CAM	N/A	*79%*		*85%*	*55%*			
Vasunilashorn 2016^46^	M, S	250	79		CAM		*24%*						
Cole 2017^47^	M, S	152	85	52	CAM	14 days			*63%*				
Reznik 2022^48^	Stroke	590	71	13	DSM-5		*48%*						

Dementia % refers to the proportion of participants in the individual study sample identified as living with dementia

% reported refer to the denominator of patients with delirium in the original sample.

M medical; S surgical; PAC post-acute care; DSM Diagnostic and Statistical Manual; CAM Confusion Assessment Method; DRS Delirium Rating Scale; DI Delirium Index

**Table 2:** Meta-regression estimating the proportion of individuals with persistent delirium

		β	Random effects estimates			After robust standard errors		
			95% CI		p	95% CI		p
Time (per month)	23 studies 39 time points	-1.6	-3.4	0.7	0.06	-4.8	1.6	0.08
Age (per year)	23 studies 39 time points	1.5	-0.4	3.3	0.12	-0.6	3.6	0.13
Dementia %	16 studies 26 time points	0.2	-0.1	0.6	0.19	-0.4	0.8	0.23

β represents % change in prevalence of persistent delirium.

Confidence intervals estimated using random-effects meta-regression (left column) and after permuting with Monte Carlo simulations (right column) Age refers to the mean age of the study sample Dementia refers to % with dementia in the study sample
