# Supplementary material for: Persistent delirium in older hospital patients: an updated systematic review and meta-analysis
Source: Delirium (Bielef). Author manuscript; Available in PMC 2023 Mar 17. (PMC7614331; doi:10.56392/001c.36822)
Supplement: Supplementary information [file EMS171842-supplement-Supplementary_information.docx]

**Supplementary Figure 1.** Assessment of risk of bias.


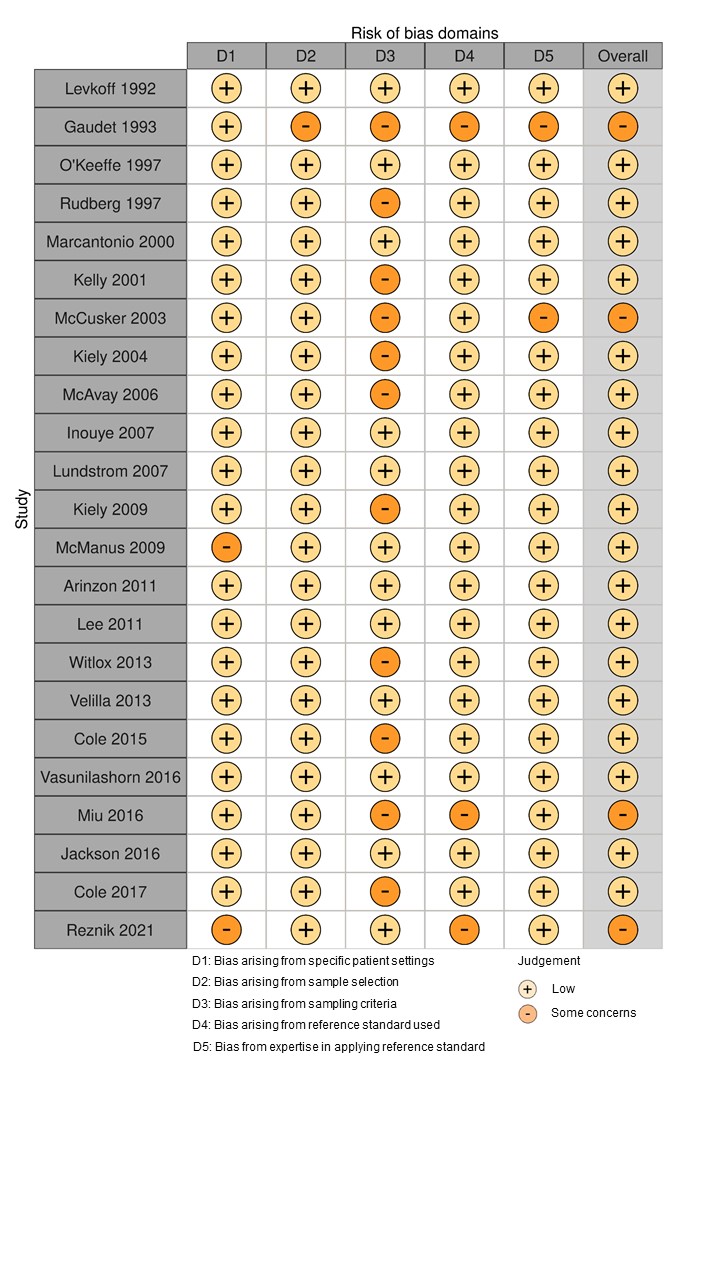


**Supplementary figure 2.** Funnel plot with pseudo 95% confidence limits.
